# Effects of Bio-Aging on Mechanical Properties and Microbial Behavior of Different Resin Composites

**DOI:** 10.3390/biom13071125

**Published:** 2023-07-14

**Authors:** Yuke Shou, Lanzhi Deng, Xiaoyu Huang, Xinyu Peng, Xinxuan Zhou, Zheng Wang, Yannan Huang, Bina Yang, Haohao Wang, Min Zhang, Lei Cheng

**Affiliations:** State Key Laboratory of Oral Diseases, National Clinical Research Center for Oral Diseases, West China Hospital of Stomatology, Sichuan University, Chengdu 610041, China; yukes@alu.scu.edu.cn (Y.S.);

**Keywords:** resin composites, bio-aging, caries prevention, mechanical properties, biofilm, microbial community

## Abstract

Under challenging oral environments, the overall performance of resin composites is affected by bio-aging. This study investigated the effects of saliva biofilm-induced bio-aging on the mechanical properties and microbial behavior of composites with different filler types. Microhybrid, nanohybrid, nano-filled and nano-filled flowable composites were bio-aged with saliva biofilm for 30 days. Surface morphology, roughness, mechanical and aesthetic properties were determined. A 48 h saliva biofilm model was used to evaluate the microbial behavior of different composites in vitro. Biofilm metabolic activity, lactic acid production and live/dead bacterial staining were tested. Six volunteers were selected to wear intra-oral appliances with composite slabs for 24 h and biofilms were collected and analyzed using 16S rRNA sequencing to assess the biofilm formation over those materials in situ. Although there were increasing trends, surface roughness, water resorption and material solubility had no significant changes for all groups after bio-aging (*p* > 0.05). There were no significant changes in elastic modulus for all groups after aging (*p* > 0.05). However, a decrease in flexural strength in all groups was observed (*p* < 0.05), except for the nanoflow composite group (*p* > 0.05). The Vickers hardness remained stable in all groups after aging (*p* > 0.05), except for the nano-filled group (*p* < 0.05). The nanoflow composite showed distinct color changes compared to the micro-hybrid group after aging (*p* < 0.05). Biofilm metabolic activity and lactic acid production in vitro increased slightly after bio-aging in all groups, but with no statistical significance (*p* > 0.05). The Shannon index diversity of biofilms in situ decreased after aging (*p* < 0.05), while no significant difference was shown in species composition at the genus level in all groups (*p* > 0.05). Resin composites with different sized fillers displayed a relatively stable mechanical performance and uncompromised microbial behavior both in vitro and in situ after 30 days of bio-aging. Based on the results, composites with different filler types can be selected flexibly according to clinical needs. However, a longer time for bio-aging is still needed to confirm the mechanical properties and microbial behaviors of composites in the long run.

## 1. Introduction

Dental caries is one of the most common oral diseases, affecting 2.3 billion adults and 520 million children worldwide [1]. The prevalence of permanent tooth caries has increased by 20.6 percent compared with the previous decade and it has caused a major public health problem across the world [2]. The main treatment for caries is restoration by removing the tooth decay and restoring teeth with materials such as resin composites and glass ionomer cement in order to stop caries progression and achieve functional recovery [3]. 

Composites have been developed into the mercury-free, environmentally friendly tooth-colored restorative materials which are commonly used in caries treatment today. Dental composites can be subdivided into macro-filled (>10 μm), micro-filled (0.01–0.1 μm) and hybrid-filled resin composites according to the particle size of their inorganic filler. With the development of nanotechnology, the particle size of fillers has progressed into the nano-scale [4]. Nanofillers are defined as inorganic filler particles with a diameter of 5–100 nanometers, typically, or aggregated as clusters with a size over 100 nm. On the nanoscale, materials exhibit very distinct properties. The application of nanoparticle fillers increases the filler content of composites, and brings the composite material obvious aesthetic and mechanical improvements [5]. In addition, the increased proportion of filler contents is capable of reducing shrinkage [6]. Moreover, by combining nanoparticles with micron fillers, the modified composites may have the advantages of both micron and nano materials [7,8,9]. However, there is still not enough evidence to prove that nano-filled or nano-hybrid composites are superior to traditional filler materials. In addition, disadvantages of nanocomposites bring concerns including reduced toughness, difficulty in controlling the particle dispersion and discrepancies in the formulations depending on the manufacturer. Compared to traditional dental composites, whether nano-filled composites have superior performance is of clinical importance and remains to be explored.

The harsh oral environment, including saliva, ions, microscopic organisms, pH value, temperature and other factors, will lead to material aging, which arises as an ongoing challenge for restorative materials. Studies have shown that the flexural strength of resin composites, giomer and glass ionomer cements decreased after aging [10]. In addition, the water absorption and dissolution expansion of aged materials could affect their surface morphology and color stability [11]. To date, most studies use physical aging models, including water aging, thermocycling aging, mechanical loading chewing simulator, and ultraviolet aging [12,13,14,15]. Those models provide a reliable evaluation of the post-aging properties of restorative materials in vitro. However, they still have certain limitations as they exclude the microbial factors, and thus can hardly simulate the complex microbiota in the oral cavity [16]. Oral microbes are considered to be one of the most challenging risk factors for restorative materials, as the microorganism degradation of polymer materials could greatly compromise the integrity of the tooth-composite margins, as well as the surface morphology [17]. Those changes could further affect the microbial behavior of materials. For example, a corroded surface with increased roughness may facilitate the early microflora colonization and biofilm accumulation and thus be more likely to cause secondary caries. Therefore, it would be highly desirable to use bio-aging models to evaluate the changes in mechanical properties and microbial behavior of composites both in vitro and in situ. 

At present, biofilm models for caries research consist of single-strain biofilms, multi-strain biofilms and saliva biofilms, among which saliva biofilms are considered to more effectively simulate the real situation of dental plaque biofilms [10,18]. In this study, human saliva microcosm biofilms were applied as a bio-aging model. After 30 days of aging, the mechanical properties of composites with different filler types were evaluated. Considering that changes in mechanical properties might, in turn, affect the biofilm accumulation on material surfaces, we further tested saliva biofilm formation on aged materials in vitro. Moreover, an intraoral device allowing saliva to freely contact the surface of specimens, forming plaque biofilms and protecting the biofilm from mechanical interference, was used to test microbiota balance in dental plaque on aged materials in situ. 

Therefore, the objectives of this study were to investigate: (1) the effects of 30 days of bio-aging on the mechanical properties of resin composites with different filler types; (2) changes in biofilm formation in vitro on resin composites before and after bio-aging; and (3) changes in the microecology balance of dental plaques in situ on resin composites before and after bio-aging. We hypothesized that mechanical properties and microbial behaviors of composites with different filler types would remain relatively stable after 30 days of aging. 

## 2. Materials and Methods

### 2.1. Resin Composites

Four kinds of commercial resin composites with different filler particle sizes were used for this study (Table 1).

### 2.2. Sample Preparation

Metallic molds with inner diameter of 10 mm and thickness of 1 mm were used as molds for round-disk samples for study in vitro. The 25 mm × 2 mm × 2 mm stain steel molds were specially manufactured for bar samples for mechanical tests. Other round-disk samples for in situ study (4 mm × 4 mm × 2 mm) were made and randomly assigned to each position in appliances as described below, fixed with silicone rubber. Briefly, composites were filled into molds and glass plates were pressed to ensure the smoothness of each surface. Light curing was applied adequately following the product instructions, exposing each side for 20 s to a curing light VALO (Ultradent Products, South Jordan, UT, USA). After demolding, excess material was carefully removed from the edges of each sample. The samples for biofilm experiments were sterilized with ethylene oxide before tests.

### 2.3. Bio-Aging with Salivary Microbe Culture

Saliva samples were collected at least 12 h after tooth brushing and 2 h after food/drink intake from 10 adult donors who met the following inclusion criteria: no systemic disease; decayed, missing and filled teeth (DMFT) <6; no periodontitis; no smoking history; and no antibiotic use within 3 months. The ethical approval was obtained from the Ethics Committee of West China Hospital of Stomatology of Sichuan University in Chengdu, China (WCHSIRB-D-2019-205). As previously described [19], the saliva samples were mixed at 1:50 volume ratio with McBain medium [20] and incubated anaerobically at 37 °C with 5% CO_2_ overnight [21,22], and then diluted to obtain 10^7^ CFU/mL saliva microbe suspension (McIntosh turbidimetric method). Temporarily unused saliva was diluted to 70% in 50% sterile glycerol, repacked in 2 mL sterile EP tubes, and frozen at −80 °C for standby.

After ethylene oxide sterilization, each round-disk sample of composites was put in each well of 24-well plates and bar samples were placed in 6-well plates. For aging, 1.5 mL/4 mL saliva microbe suspension was added in each well of 24-well/6-well plates and similarly incubated anaerobically at 37 °C with 5% CO_2_ for 30 days. McBain medium was replaced every 24 h. Afterwards, the bacterial biofilms on the sample were washed through ultrasonic vibration and the samples were naturally dried for subsequent experiments. 

### 2.4. Scanning Electron Microscopy (SEM) Observation

To characterize the surface configuration of the composites under SEM (Quanta 200, FEI, Hillsboro, OR, USA), the round-disk samples were rinsed with phosphate buffered saline (PBS) twice, fixed with 2.5% glutaraldehyde overnight, serially dehydrated with ethanol (50%, 60%, 70%, 80%, 90%, 95%, and 100%), and then sprayed with a thin layer of gold. During observation, three fields were randomly determined for each sample to obtain SEM images [10]. Six samples were tested for each group.

### 2.5. Atomic Force Microscope (AFM) Observation

To analyze the surface properties of composites, AFM (5500 SPM, Agilent, Santa Clara, CA, USA) was used to identify the 3-dimensional (3D) morphology and roughness parameters of samples before and after aging. High resolution with a sharp silicon tip and peak force tapping model was set for AFM tests. Three fields of each sample were randomly chosen to acquire surface topography and surface average roughness (Ra) values [23]. The 3D reconstructions of surface morphology were accomplished with systemic software (SPIWIN 2.0, Seiko, Tokyo, Japan). Six round-disk samples were tested for each group. 

### 2.6. Flexural Strength and Elastic Modulus Tests

The flexural strength and elastic modulus of composites were evaluated through three-point bending tests using 25 mm × 2 mm × 2 mm bar samples, with loading conditions of 20-mm spans and crosshead speed of 1 mm/min on a computer-controlled Universal Testing Machine (5500 R, MTS, Cary, NC, USA). The load and displacement values were recorded until the breaking of the samples.

Flexural strength δ (MPa) calculation formula was δ = 3FL/(2BH^2^), where F is the maximum load when the resin specimen is damaged (N), L is the span (mm), B is the width of the samples (mm) and H is the height of the samples (mm). The calculation formula of elastic modulus E (GPa) was E = (F/D) (L^3^/[4BH^3^])/1000, where F (N) is the load in the linear elastic zone, D (mm) is the displacement in the linear elastic zone, F/D (N/mm) is the gradient of the linear part of the load-displacement curve, l is the span (mm), B is the width of the samples (mm) and H is the height of the samples (mm) [24]. Eight bar samples were tested for each group.

### 2.7. Vickers Hardness Measurement Using Indentation Method

Indentation was applied on the experimental surface of the round-disk samples with digital Vickers hardness tester HVS-50. The samples were placed under the regular pyramid diamond indenter (the angle between the two opposite sides of the indenter was 136°) with the loading force of 98 N for 15 s. Five points at different areas of each sample were tested. The diagonal length of each indentation on the test surface was measured twice under the inverted metallographic microscope to take the average value [25]. Vickers hardness (HV) was calculated according to the following formula: HV = 1.854 P/(2a)^2^, where HV: Vickers hardness (MPa), P: indenter load (N, 98N here) and 2a: indentation diagonal length (mm). Six samples were tested for each group, respectively.

### 2.8. Measurement of Water Resorption and Solubility

These mechanical measurements were based on International Standard Organization (ISO) Specification No. 4049. Round-disk samples were first dried in the drying box at 37 °C for 22 h, and then placed in the drying box at 23 °C for 2 h. The samples were weighed on the analytical balance [26]. The above procedures were repeated until the difference between the two successive mass measurements was less than 0.1 mg and the current mass was recorded as *M1*. When drying was finished, the diameter and thickness of each sample were measured with a vernier caliper to calculate the volume *V* of the samples. The samples were then soaked in 15 mL distilled water in plastic bottles in a 37 °C water bath for 7 days. After drying the excess water on the surface with filter paper, the samples were weighed again on the analytical balance and the mass was recorded as *M2*. After that, the drying procedures were repeated and the ultimate mass was recorded as *M3*. Six round-disk samples were tested for each group. 

The water resorption of composites was calculated as follows: WR = (*M2* − *M3*)/*V*. The solubility of composites was calculated as follows: SL = (*M1* − *M3*)/*V*. Six round-disk samples were tested for each group.

### 2.9. Colorimetry Tests

A specialized dental color analysis system, Crystaleye Spectrophotometer (Olympus, Tokyo, Japan), was utilized to measure the color difference (ΔE) of samples before and after aging. Zero-correction was conducted with the standard black box and standard white ceramic tile and standard light source D65 was calibrated in reflection mode before testing. According to the CIELAB chromaticity system, all colors can be defined by the coordinates of the three axes L*, a*, and b*, which, respectively, represent lightness, the chromaticity coordinate in a red–green direction and the chromaticity coordinate in a yellow–blue direction. The black standard ceramic tile was taken as the background-disk, and the color parameters L*, a*, b* of the three randomly selected regions of each sample were measured with the spectrophotometer in Crystaleye Spectrophotometer. The differences of three coordinates between the values before and after aging were defined as ΔL*, Δa* and Δb*. The total color differences were calculated following the formula ΔE = [(ΔL*)^2^ + (Δa*)^2^ + (Δb*)^2^]^1/2^. Six round-disk samples were tested for each group, respectively [27].

### 2.10. Biofilm Cultivation

To estimate the microbial behavior changes in composites before and after aging, saliva biofilms were grown on round-disk samples in vitro [10]. The saliva from donors was revived by sitting on ice and mixed with McBain medium at the volume ratio of 1:50 to obtain the inoculums of salivary microbes. The sterilized samples were placed in a 24-well, as previously described, and 1.5 mL inoculum solution was added in each well. The salivary microbes were cultivated for 48 h with the renewal of McBain medium at 8 h and 24 h, respectively.

### 2.11. Lactic Acid Production Measurement

To measure the acid-producing ability of the attached biofilms, samples with 48 h biofilms were washed with cysteine peptone water (CPW) gently to remove unattached bacteria, then immersed in 1.5 mL buffered peptone water (BPW, Sigma-Aldrich, St. Louis, MO, USA) supplemented with 0.2% sucrose. The biofilms were next incubated at 37 °C with 5% CO_2_ for 3 h. Then, the culture medium BPW was collected for lactate analysis using the lactate dehydrogenase enzymatic method [28]. Following the instructions of Lactic Acid assay kit (Nanjing Jiancheng Bioengineering Institute, Nanjing, China), OD_340 nm_ of each sample was compared to the lactic acid standard curves formed by serial OD_340 nm_ values at concentrations of 0, 0.5, 1, 1.5, 2, 2.5, 3 and 3.5 mM. Six samples were tested for each group.

### 2.12. MTT Metabolic Assay

Since the enzymes from living bacteria can reduce 3-(4,5-Dimethylthiazol-2-yl)-2,5-diphenyltetrazolium bromide (MTT) to water-insoluble purple formazan, the metabolic activity of saliva biofilms can be evaluated by MTT assay. Round-disk samples with 48 h biofilms were washed with PBS and transferred into a new 24-well plate. Then, 1 mL MTT solution (0.5 mg/mL MTT in PBS) was added into each well and incubated at 37 °C with 5% CO_2_ for 1 h in a dark place [29]. After discarding the supernatant, the samples were transferred into a new 24-well plate on a shaking platform with 1 mL dimethyl sulfoxide (DMSO) in each well to dissolve the formazan crystals [30]. After 20 min, 200 μL of the solution in each well was transferred into 96-well plates and examined in a microplate reader (Gene, Hong Kong, China) for OD_540 nm_. Six samples were tested for each group.

### 2.13. Live/Dead Bacterial Staining

The cultured biofilms were rinsed gently in PBS and stained following the instructions of Live/Dead Bacterial Fluorescence Kit (Invitrogen, Carlsbad, CA, USA). The living bacteria stained with SYTO 9 produced green fluorescence, while dead bacteria with impaired cell membranes exhibited red fluorescence colored by sodium propionate iodide. The samples were observed under confocal laser scanning microscope (FV100, Olympus, Shinjuku City, Japan) equipped with a 60 × oil immersion objective lens. The adjacent or overlapping live and dead bacteria showed orange or yellow color [31]. The area fraction of live bacteria green staining area/dead bacteria red staining area of image was measured using ImageJ 1.48 v (National Institutes of Health, Bethesda, MD, USA). All three-dimensional reconstructions of the in vitro biofilms were performed using Imaris 7.0.0 (Bitplane, Zürich, Switzerland). Three samples were evaluated in each group, and three fields were randomly selected from each sample. 

### 2.14. Design of Palatal Appliance and Plaque Collection

An in situ oral biofilm collection device was applied as previously described [19]. The device was made of adelomorphic retainer materials. Each volunteer had their own custom oral device. Each device could be equipped with 6 disc-shaped samples (D = 4 mm, H = 2 mm). The samples were placed in the sample tank at the jaw arch of the device. Each sample tank had holes on the surface, allowing saliva to freely contact the sample surface, forming plaque biofilm and protecting it from mechanical interference.

This study was approved by ethical review, as previously described. Six healthy adult volunteers were recruited, with natural dentition, no systemic diseases, no smoking, low caries (0 < DMFT < 6), no allergic history of dental and oral hygiene materials, and no use of antibiotics, steroids and other drugs that may cause xerostomia in the last 3 months. Participant wore the device for more than 20 h a day. The devices were removed only when eating meals and brushing teeth, and stored in sterile PBS. The 24 h biofilms collected from the facility in situ were immediately DNA extracted or, if necessary, frozen and stored at −80 °C until further analysis. Up to six samples could be installed in each in-situ appliance. For each participant, two specimens of each material were set up for sequencing.

### 2.15. Gene Sequencing

The biofilms samples were subjected to Majorbio Co. Ltd. (Shanghai, China) and the total microbial genomic DNA was extracted and sequenced according to manufacturer procedures. As previously described [32], DNA was isolated using an E.Z.N.A.^®^ soil DNA Kit (Omega Bio-tek, Norcross, GA, USA). The DNA concentration was determined using a NanoDrop spectrophotometer (Thermo Scientific, Wilmington, DE, USA). The hypervariable region V4–V5 of the bacterial 16S rRNA gene was amplified with 515F 907R barcoded primer pairs with an ABI GeneAmp^®^ 9700 PCR thermocycler (ABI, Vernon, CA, USA). The PCR amplification of the 16S rRNA gene was performed in triplicate. PCR mixtures contained 5 × TransStart FastPfu buffer 4 μL, 2.5 mM dNTPs 2 μL, forward primer (5 μM) 0.8 μL, reverse primer (5 μM) 0.8 μL, TransStart FastPfu DNA Polymerase 0.4 μL, template DNA 10 ng and finally ddH2O up to 20 μL. The PCR amplicons were extracted from 2% agarose gel and purified with the AxyPrep DNA Gel Extraction Kit (Axygen Biosciences, Union City, CA, USA) and quantified using a Quantus™ Fluorometer (Promega, Madison, WI, USA). Purified products were pooled in equimolar and paired-end sequenced using an Illumina MiSeq PE300 platform (Illumina, San Diego, CA, USA) according to the standard protocols by Majorbio (Shanghai, China). The raw reads were deposited into the NCBI Sequence Read Archive (SRA) database (BioProject ID: PRJNA908123), then demultiplexed, quality-filtered with fastp version 0.20.0 and merged with FLASH version 1.2.7 with the following criteria: (i) the 300 bp reads were truncated at any site receiving an average quality score of <20 over a 50 bp sliding window, and the truncated reads shorter than 50 bp were discarded, and reads containing ambiguous characters were also discarded; (ii) only overlapping sequences longer than 10 bp were assembled according to their overlapped sequence. The maximum mismatch ratio of overlap region was 0.2. Reads that could not be assembled were discarded; (iii) Samples were distinguished according to the barcode and primers, and the sequence direction was adjusted, exact barcode matching, 2 nucleotide mismatches in primer matching.

Operational taxonomic units (OTUs) with 97% similarity cutoff were clustered using UPARSE version 7.1, and chimeric sequences were identified and removed. The taxonomy of each OTU representative sequence was analyzed witha RDP Classifier version 2.2 against the 16S rRNA database (Silva Release 138, http://www.arb-silva.de, accessed on 24 April 2022) using a confidence threshold of 0.7. Alpha diversity indices (Shannon index), and richness estimator (ACE index) calculations were performed using Mothur v.1.30.2. Phylogenetic beta diversity measures such as the unweighted UniFrac distance metrics analysis were determined using the represent sequences of OTUs for each sample, and principal component analysis (PCA) was conducted according to the distance matrices. The data of donor and dental plaque samples were not involved in any statistical analysis.

### 2.16. Statistical Analysis

The data were statistically analyzed using the SPSS software, version 16.0 (SPSS Inc., Chicago, IL, USA). One-way analysis of variance (ANOVA), Tukey’s multiple posttests or the Wilcoxon rank sum test were used to detect the significant effects in different groups before and after aging with the level of statistical significance set at *p* < 0.05.

## 3. Results

### 3.1. SEM and AFM Observation

**Figure 1:**
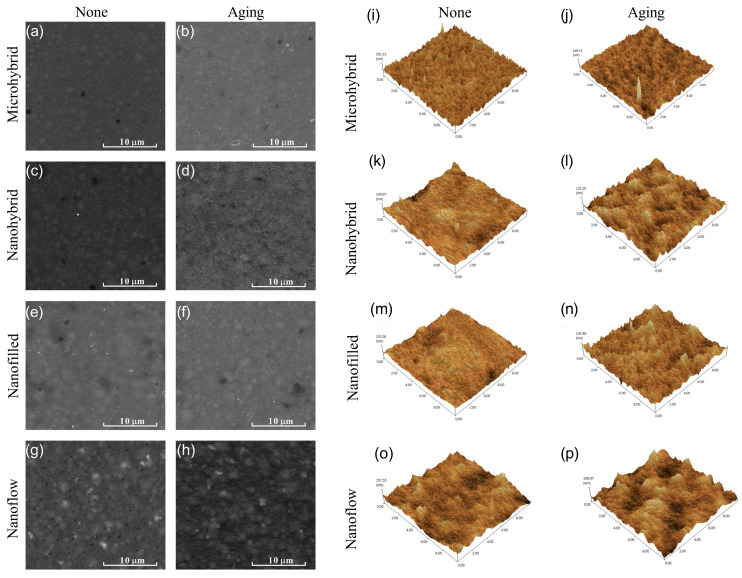
Representative SEM images of composites before (**a**,**c**,**e**,**g**) and after (**b**,**d**,**f**,**h**) 30-day bio-aging. Representative AFM images of composites before (**i**,**k**,**m**,**o**) and after (**j**,**l**,**n**,**p**) 30-day bio-aging.

**Figure 2:**
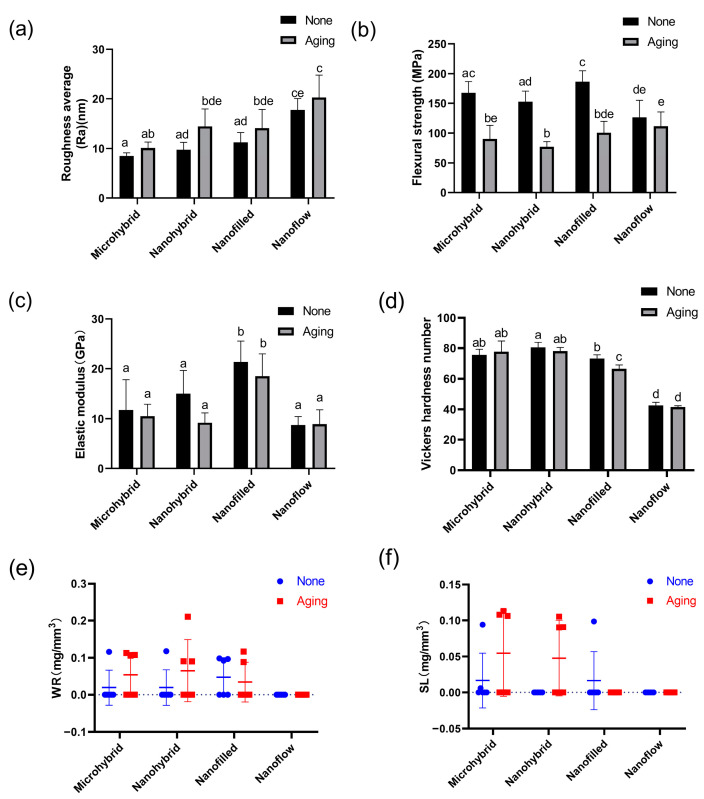
Statistical analysis of roughness average (**a**), flexural strength (**b**), elastic modulus (**c**), Vickers hardness (**d**), water resorption (**e**) and solubility (**f**) of composites before and after 30-day bio-aging. Each value is mean ± SD; *n* = 6. Values with dissimilar letters are significantly different from each other (*p* < 0.05).

The representative SEM images of composites before and after aging are shown in Figure 1a–h. The surface morphology of the same sample before and after aging is shown by AFM images in Figure 1i–p and by the statistical analysis of roughness average (Ra) in Figure 2a. Among untreated samples (Figure 1i,k,m,o and Figure 2a), the nanoflow composite showed a greater Ra than other groups (*p* < 0.05). The aging treatment with salivary microbes for 30 days seemed to increase the surfaces’ roughness, but with no statistical significance (*p* > 0.05). Comparing the four composites after aging (Figure 1j,l,n,p and Figure 2a), micro-hybrid composites exhibited the smoothest surfaces, followed by nano-hybrid and nano-filled ones, while the nanoflow composite showed the roughest. 

### 3.2. Mechanical Properties

The results of flexural strength and elastic modulus are shown in Figure 2b,c. The aging treatment caused a significant decrease in the flexural strength in all groups (*p* < 0.05) except for the nanoflow composite group (*p* > 0.05). There were no significant changes in the elastic modulus for all groups after aging (*p* > 0.05). The Vickers hardness values of four composites before and after aging are shown in Figure 2d. The nanoflow composite showed the lowest hardness among all the groups (*p* < 0.05). After aging, a significant hardness decrease was only seen in the nano-filled group (*p* < 0.05). For water resorption and solubility, there was no significant difference before and after aging in all groups (*p* > 0.05). All groups showed good resistance to water absorption and dissolution under bio-aging challenges (Figure 2e,f).

### 3.3. Colorimetry Tests

**Figure 3:**
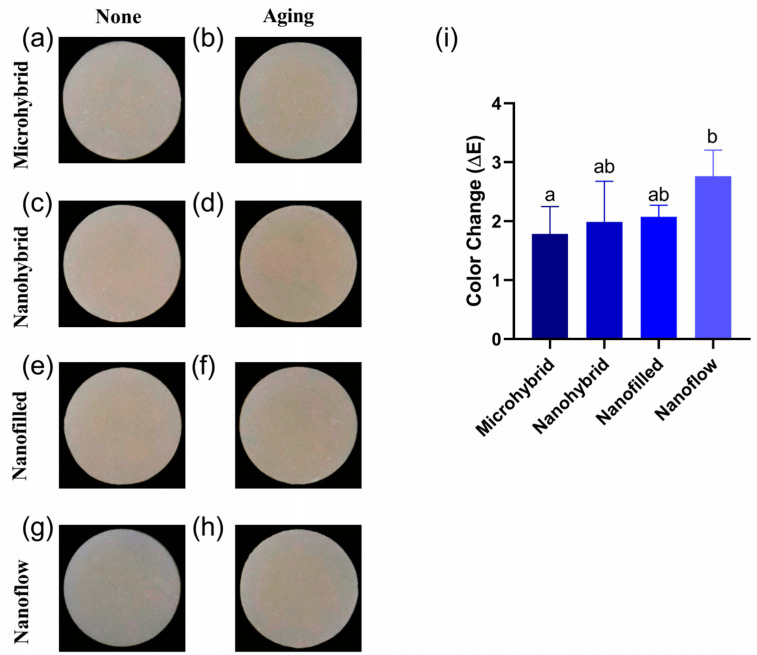
(**a**–**h**) Representative photos of composites before (**a**,**c**,**e**,**g**) and after (**b**,**d**,**f**,**h**) 30-day bio-aging. (**i**) Color changes (ΔE) of four tested materials after 30-day bio-aging. All the ΔE values were obtained by comparing to values of initial samples before aging. Each value is mean ± SD, *n* = 6. Values with dissimilar letters are significantly different from each other (*p* < 0.05).

The results of the colorimetry tests before and after aging were shown in Figure 3. Figure 3a–h exhibit the photos taken with the Crystaleye Spectrophotometer for colorimetry tests. The statistical analysis of color changes in each group were shown in Figure 3i. The color changes of the four materials were all considered acceptable, based on the clinically acceptable values of ΔE < 3.3. The nanoflow composites showed the most obvious color change after aging (*p* < 0.05).

### 3.4. Lactic Acid Production Measurement and MTT Assay

**Figure 4:**
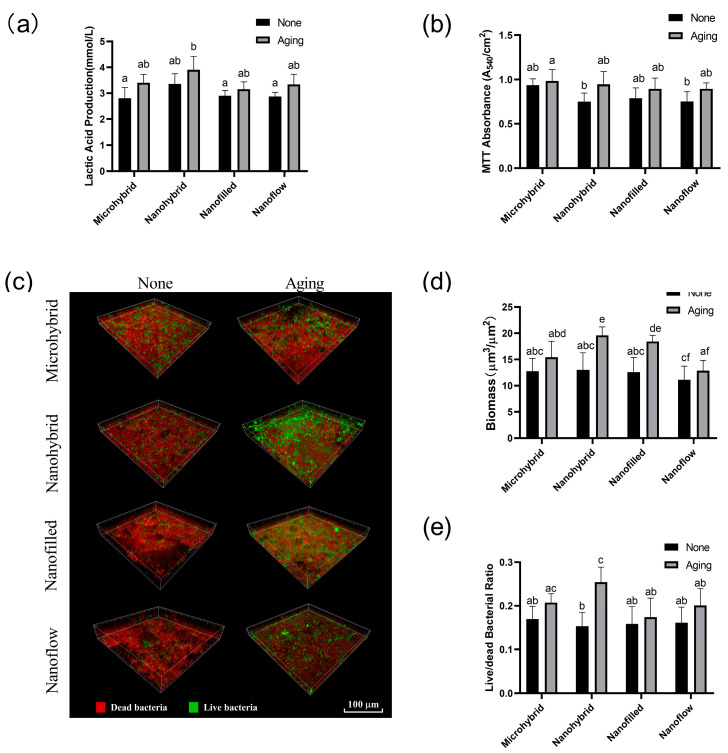
(**a**) Lactic acid productions of 48 h saliva biofilms on composites before and after aging. (**b**) The MTT metabolic activity of 48 h saliva biofilms on composites before and after aging. (**c**) Live/dead bacterial staining of the 48 h saliva biofilms on composites before and after 30-day bio-aging. Statistical analysis of biomass (**d**) and live/dead bacterial ratio (**e**) of the 48 h saliva biofilms on composites before and after 30-day bio-aging. Each value is mean ± SD, *n* = 6. Values with dissimilar letters are significantly different from each other (*p* < 0.05).

The results of the lactic acid production measurement and MTT assay are shown in Figure 4a,b. Biofilms on all the groups had similar metabolic activity and acid-producing ability before and after the 30-day bio-aging. The activity of biofilm metabolism and lactic acid production in each group showed an increasing trend after aging, but there was no significant difference (*p* > 0.05).

### 3.5. Live/Dead Bacterial Staining

The live/dead bacterial staining images of biofilms are shown in Figure 4c. The biomass of biofilms and viability of bacteria calculated by the fluorescence statistics are shown in Figure 4d,e. Biofilm volumes on aged samples increased compared to those on initial samples before aging, especially in nanohybrid and nano-filled groups (*p* < 0.05), as shown in Figure 4d. The aging treatment significantly raised the ratio of live bacteria on nanohybrid composites (*p* < 0.05), as shown in Figure 4e, while no significant change was observed in the other three groups before and after aging (*p* > 0.05).

### 3.6. 16S rRNA Gene Sequencing

**Figure 5:**
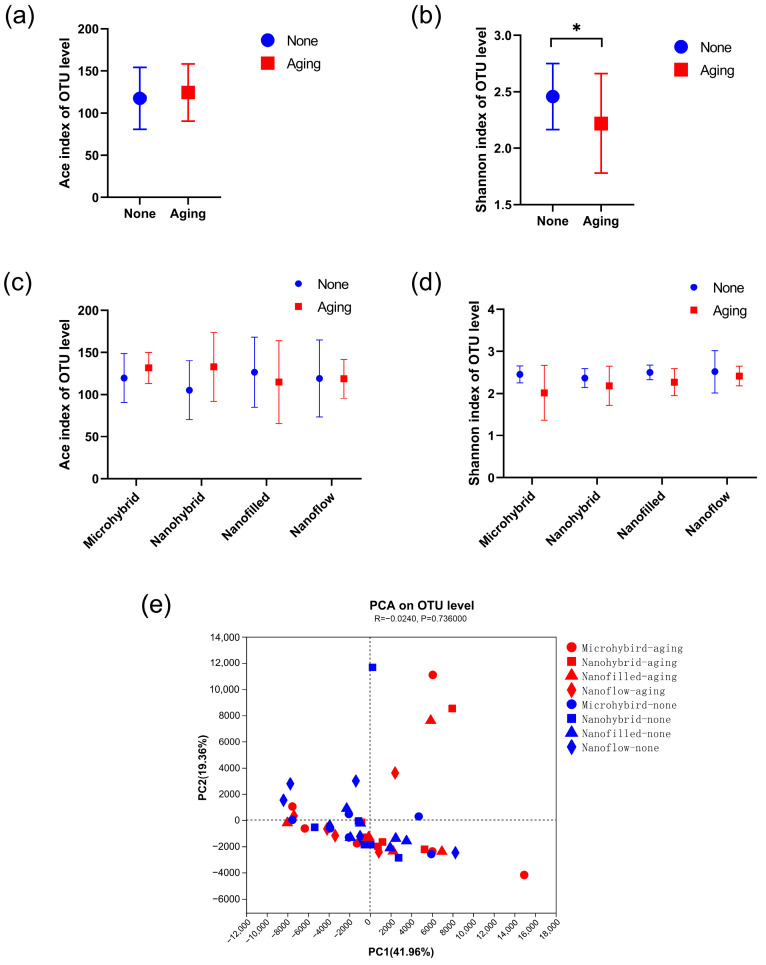
The alpha and beta diversity. (**a**–**d**) Alpha diversities based on OTUs: (**a**) shows the total richness of four composites as a whole group before and after aging by Ace and the total diversity by (**b**) Shannon indices, while (**c**,**d**) show the richness and diversity by the Ace and Shannon indices of different composite groups. The Kruskal–Wallis test was used to compare the significant difference. (**e**) Beta diversity by PCA of all groups based on the distance matrices. PC1 and PC2 explained 61.32% of the total PCA variability. A *p*-value of < 0.05 was considered statistically significant (* *p* < 0.05).

**Figure 6:**
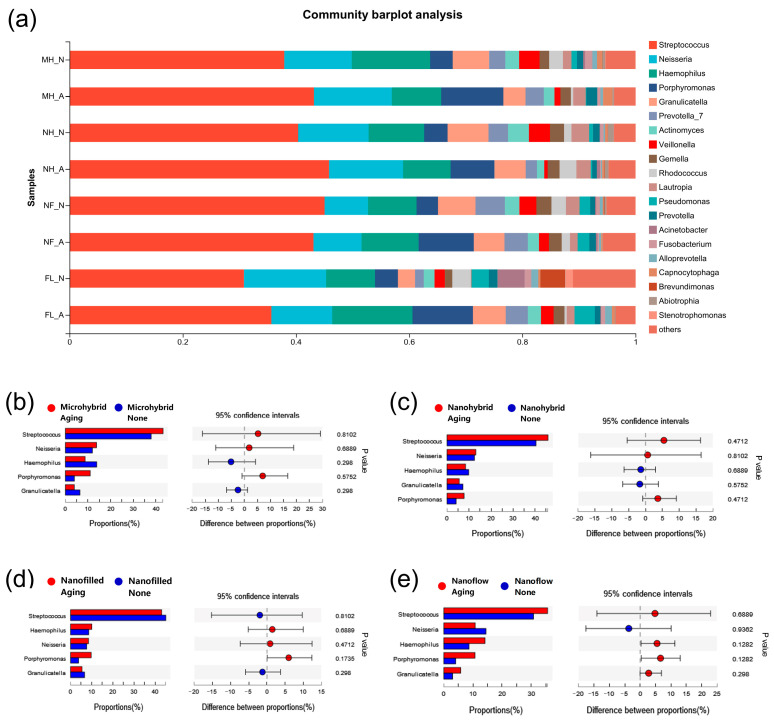
The proportion of community abundance at genus level of the 24-h in situ biofilms. (**a**) The comparisons of biofilms on composites before and after aging at genus level. (**b**–**e**) The other data subjected to Wilcoxon rank-sum test on genus level. A *p*-value of < 0.05 was considered statistically significant.

The results of the α diversity of the 24-h in-situ oral biofilms showed that the ACE index had a trend of increasing after aging, while the Shannon index trend was to decrease (Figure 5a,b), indicating an increasing trend in biofilm richness and a decreasing trend in diversity. However, significantly, a difference was found only when comparing the Shannon index of the four groups as a whole before and after aging (Figure 5b), while there was no significant difference in the comparison within each composite group before and after aging (Figure 5c,d). As for β diversity analysis, the Principal Component Analysis (PCA) exhibited clusters of the microbial community composition before and after aging (Figure 5e). The proportion of relative abundance of each dominant genus is shown in Figure 6a. The assessments of the significance of abundance differences at genus level disclosed the microbial community characteristics of the collected in situ biofilms. *Streptococcus*, *Neisseria*, *Haemophilus*, *Porphyromonas* and *Granulicatella* were the majority in all groups, and no significant differences were shown among these dominant genera (Figure 6b–e). 

## 4. Discussion

This study investigated the effects of 30 days of saliva microbial aging on mechanical properties of composites with different filler types, as well as changes in the biofilm formation and microecology balance on composites before and after bio-aging. Four commercial composites with micro-hybrid, nano-hybrid, nano-filled and nano-flowable fillers were selected in order to study whether a nanoscale upgrade of the filler particle size would lead to clinically desirable improvements. A saliva biofilm-induced aging model was used to better simulate the real oral environment by incorporating the microbial factors during aging. After bio-aging, the surface morphology had slight changes in all groups, showing a more uneven appearance and granular pits on the material surface. However, the changes in the surface average roughness were not significantly different in all groups, suggesting that the structural changes on material surfaces under 30 days of bio-aging challenge were not enough to cause an increase in roughness. Fracture is one of the main causes of restoration failures, and it is closely related to the mechanical properties of restorative materials against aging. In our study, although there was a decreasing trend, all composites maintained a stable elastic modulus after 30 days of bio-aging. However, the flexural strength decreased significantly in most groups, which was consistent with many previous studies. For example, studies of various low shrinkage commercial composites found that the flexural strength was significantly reduced after 6 months and 1 year of water aging [10,33]. Oliveira used a mechanical loading chewing simulator to test the mechanical properties of the 3M350 composite and bulk-fill composite after 5 years of use. Similarly, both materials were significantly weakened in flexural strength after aging [13]. In terms of surface hardness, the nanoflow composite showed the lowest Vickers hardness and is therefore the least suitable to use in stress concentration areas, such as the occlusal surface. Only the nano-filled group exhibited a significant decrease in the surface hardness after aging, which may be related to the fact that the agglomerated structure of nanofillers is more loose than monolithic micromaterials, and the matrix infiltrated in the tiny gaps of nanofillers can be degenerated under the long-term effect of organic acids, resulting in a greater degree of hardness drop [7]. Nonetheless, studies have shown that a higher nanoparticles ratio can improve the mechanical properties of materials in aspects of polymerization shrinkage. With the incorporation of nanoparticles, the polymerization shrinkage and sidewall stress of composites can be reduced without changing other essential properties [34]. However, the optimum weight percentage of nanofillers, around 20% wt, should not be exceeded as the mechanical properties will not be further improved after this point [7], indicating that nanohybrid materials may have better mechanical potential than nano-filled materials. In terms of water absorption and solubility, all materials showed a reassuring performance after 30-day aging. But further studies with longer-term aging challenges are needed, as high-molecular polymers are prone to water absorption and swelling due to the reduction in the bonding strength between polymer chains [35,36].

The color of restorations and their stability after long-term use are important factors in the selection of dental materials. Most previous studies on the color changes of restorations used water aging models without considering the aging effects from microbes [37,38]. However, it can be speculated that the accumulation of organic acids, enzymes and other microbial metabolites can affect the color performance of restorations. Therefore, in this study, we used a saliva biofilm-induced aging model to introduce the microbial factor. The color change of composites before and after bio-aging was rarely observed with naked eyes. Then, the CIE color difference formula was used to quantify the color change ΔE before and after aging. The results showed that the color change of the nanoflow composite was most obvious, but still clinically acceptable, at ΔE < 3.3, while the micro-hybrid composite exhibited the most stable color with the lowest ΔE. Overall, the aesthetic properties of composites with different filler types showed clinically acceptable stability after 30 days of bio-aging.

Degradation and structural changes on the material surfaces may affect the acquired membranes formation and early bacterial attachment, thus affecting subsequent biofilm formation. Therefore, we tested the biofilm on composites before and after 30-day bio-aging in vitro. The biomass and proportion of live bacteria in the biofilm on aged composites increased slightly, but most of it was not significantly different. In addition, metabolic indicators such as metabolic activity and acid production did not increase significantly after aging, either. This may be due to the results showing that the surface roughness of all groups remained relatively smooth after aging, which were all below 30 nm, as previous studies revealed that when the surface roughness of materials is below 200 nm, a further decrease in roughness could not promote the reduction of bacterial attachment and biofilm formation [39,40]. Therefore, with appropriate polishing, the roughness of all composite groups can be smoothed enough regardless of the filler type and composition. Although it is generally believed that the surface roughness is an important factor affecting biofilm formation on materials, the relationship between roughness and microbial attachment might also be related to the components and characteristics of the material itself [41], and general concepts of threshold are not applicable to all materials [42]. A consensus view is that polishing strategies should be taken to make the material surfaces as smooth as possible at the end of treatments. 

Dental plaque is a diverse community composed of hundreds of microorganisms in a dynamic equilibrium, and caries happen when the equilibrium is broken down by the overgrowth of acid-tolerant species and acid-producing species outcompeting the commensal microbiota, leading to a microbiome dysbiosis and a reduction in microbial diversity [43]. Previous studies have shown that the degradation of resin polymers contributes to increase the cariogenic activity of bacteria and increase the formation of cariogenic biofilms, which in turn accelerates resin aging and biodegradation, forming a vicious cycle [44,45]. Therefore, the effects of aging on the microbiota balance in biofilms on composites became a concern for us. We used an in-situ biofilm model to study the Alpha and Beta diversities of plaques on composites before and after bio-aging. Biofilms on composites of each group showed a decreasing trend in diversity after aging, but with no significant difference. Only when biofilms of the four composites were analyzed as a whole group did the Shannon index diversity show a significant decrease after aging. We further measured the proportion of community abundance at genus level in each group. All groups shared a similar species composition, with *Streptococcus*, *Neisseria*, *Haemophilus*, *Porphyromonas* and *Granulicatella* dominating the biofilms. Notably, the abundance of *Porphyromonas* had an increasing trend in all aged groups, but with no significant difference. *Porphyromonas* is a key component of plaque biofilm and plays a vital coordination role between the early and late colonizing bacteria of dental biofilms [46]. If this trend continues to increase, it may eventually lead to changes in the microecological balance of co-aggregates and late colonizers in mature plaque biofilm. Therefore, the effect of longer-term aging challenges on the microbial behavior of composites needs to be further studied. Unlike most laboratory studies, we included an in-situ biofilm model to reproduce the aging process in the oral cavity. This method improved the comparability to real conditions, and these conclusions may likely be extrapolated to the clinical situation. Nevertheless, since the oral cavity is a complex, ever-changing world, the salivary biofilm model used in this laboratory study may not accurately simulate the clinical situation. It has some limitations, including its laboratory conditions and the selection of limited materials and procedures. We should acknowledge the discrepancy between laboratory and clinical research, and the results of this study should be corroborated by long term controlled clinical studies.

## 5. Conclusions

This study investigated the effects of aging on the mechanical properties and microbial behaviors of composites with different filler types using a saliva biofilm-induced aging model for 30 days. In terms of mechanical properties, the surface roughness, water resorption and material solubility of composites had no significant difference after bio-aging. The nanoflow composite showed the most obvious color change after aging, but it was still clinically acceptable. As for microbial behaviors, biofilm metabolic activity and lactic acid production showed an increasing trend, but with no significant difference. Biofilm volumes in vitro on aged samples increased compared to those on initial samples before aging, especially in nano-hybrid and nano-filled groups. The aging treatment significantly raised the ratio of live bacteria on nanohybrid composites, while no significant change was observed in the other three groups. The in situ oral biofilm collection device played an important role in the detection of microecological balance in dental plaque on composites. The Shannon index diversity of biofilms in situ decreased after aging. No significant difference was shown in the composition of biofilms on composites before and after aging at genus level. These results indicate that properties of composites with different filler types remain relatively stable after 30-day aging. However, when applying to aesthetic areas, paste composites, according to our results, seem to be more suitable for maintaining a stable color against aging. At other times, they can be selected flexibly according to clinical needs. Nevertheless, a longer time for bio-aging is still needed to confirm the mechanical properties and microbial behaviors of composites in the long run.

## Figures and Tables

**Table 1:** Material information ^1^.

Product Name	Manufacturer	Resin Base	Shade	Filler Chemistry	Filler Particle Size	Material Type
Filtek™ Z250	3M ESPE, Irvine, CA, USA	Bis-GMA, UDMA, Bis-EMA	A2	Zirconia and Silica	0.01–3.5 μm	Micro-hybrid
Filtek™ Z250 XT	3M ESPE, Irvine, CA, USA	Bis-GMA, UDMA, Bis-EMA, TEGDMA	A2	Zirconia and Silica	0.02–3.5 μm	Nano-hybrid
Filtek™ Z350 XT	3M ESPE, Irvine, CA, USA	Bis-GMA, UDMA, TEGDMA, Bis-EMA	A2	Zirconia and Silica	4–20 nm	Nano-filled
Filtek™ Z350 XT Flowable Restorative	3M ESPE, Irvine, CA, USA	Bis-GMA, UDMA, TEGDMA	A2	Ytterbium Trifluoride, Zirconia and Silica	4 nm–5 μm	Nanoflow

^1^ The resin composites with different filler particle sizes used in this study are shown in Table 1.

## Data Availability

The data that support the findings of this study are available from the corresponding author, H.W., upon reasonable request.
